# PGK1 and GRP78 overexpression correlates with clinical significance and poor prognosis in Chinese endometrial cancer patients

**DOI:** 10.18632/oncotarget.23090

**Published:** 2017-12-07

**Authors:** Suiqun Guo, Yanyi Xiao, Danqing Li, Qingping Jiang, Litong Zhu, Dan Lin, Huiping Jiang, Wei Chen, Lijing Wang, Chunhua Liu, Weiyi Fang, Li Lin

**Affiliations:** ^1^ Department of Obstetrics and Gynecology, The Third Affiliated Hospital of Southern Medical University, Guangzhou, 510630, P.R. China; ^2^ Cancer Center, Integrated Hospital of Traditional Chinese Medicine, Southern Medical University, Guangzhou, Guangdong, 510315, P.R. China; ^3^ Department of Pathology, Third Affiliated Hospital of Guangzhou Medical College, Guangzhou, 510150, P.R. China; ^4^ Department of Healthy Management, Nanfang Hospital, Southern Medical University, Guangzhou, Guangdong, 510515, P.R. China

**Keywords:** endometrial carcinoma, phosphoglycerate kinase 1, glucose-regulated protein 78, immunohistochemistry, clinicopathological characteristics

## Abstract

The aim of this study was to measure the expression patterns of PGK1 and GRP78 in normal endometrial tissues and endometrial carcinoma, and associations between their combined effects and the pathological features of endometrial carcinoma. We used 30 normal endometrial tissue samples and 130 endometrial carcinoma samples, and separately evaluated PGK1 and GRP78 protein expression by immunohistochemistry. Scores ranging from 0 to 9 were obtained by multiplying the percentage of positive cells by the staining intensity (0–3). Immunohistochemical analysis revealed increased PGK1 and GRP78 expression in the cytoplasm of endometrial carcinoma cells compared with that in normal endometrial tissues. High PGK1 expression positively correlated with the FIGO stage (*P* < 0.001), histological grade (*P* = 0.002), and lymph node status (*P* < 0.001). High GRP78 expression positively correlated with the pathological type (*P* = 0.0125), FIGO stage (*P* < 0.001), and lymph node status (*P* < 0.001). In addition, PGK1 overexpression was positively correlated with GRP78 overexpression in endometrial carcinoma patients (*P* < 0.001), and the concurrent expression of both oncogenes in endometrial carcinoma patients correlated significantly with the lymph node status (*P* < 0.001) and FIGO stage (*P* < 0.001). Patients with high PGK1 and GRP78 expression levels had poorer overall survival rates than those with low expression levels of both proteins (*P* < 0.001). Our results suggested that the co-occurrence of PGK1 and GRP78 expression is potentially an unfavorable factor for endometrial carcinoma progression.

## INTRODUCTION

Endometrial cancer is the most common gynecological tumor in developed countries, and its incidence continues to increase [1]. Despite improved overall survival rates, the incidence of endometrial carcinoma has risen by 40% over the past 20 years and associated deaths have risen by 20%, primarily because its etiopathogenesis is complicated and not fully understood [2]. Understanding pathogenesis at the molecular level is essential for identifying useful biomarkers for use in targeted therapies [3].

**Figure 1:**
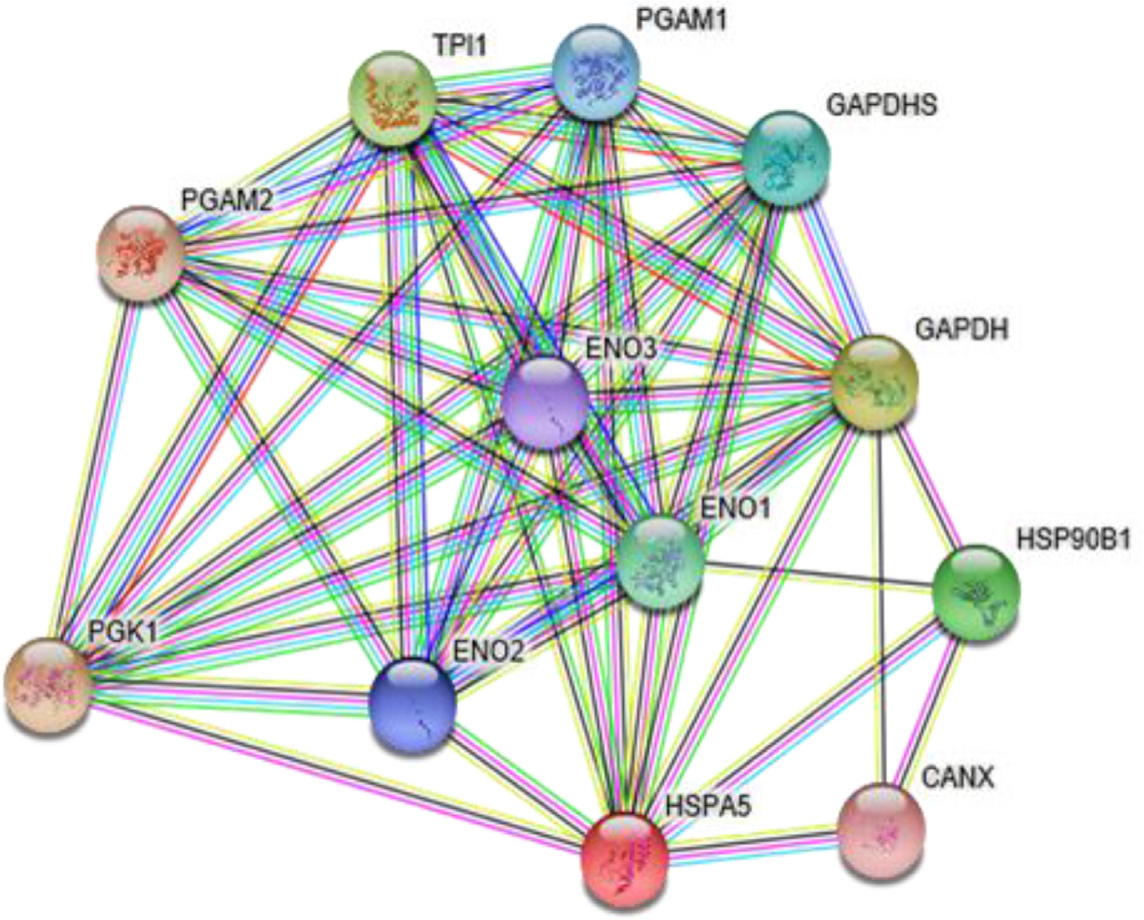
The related genes in a network of glucose metabolism, as predicted using the STRING network

**Figure 2:**
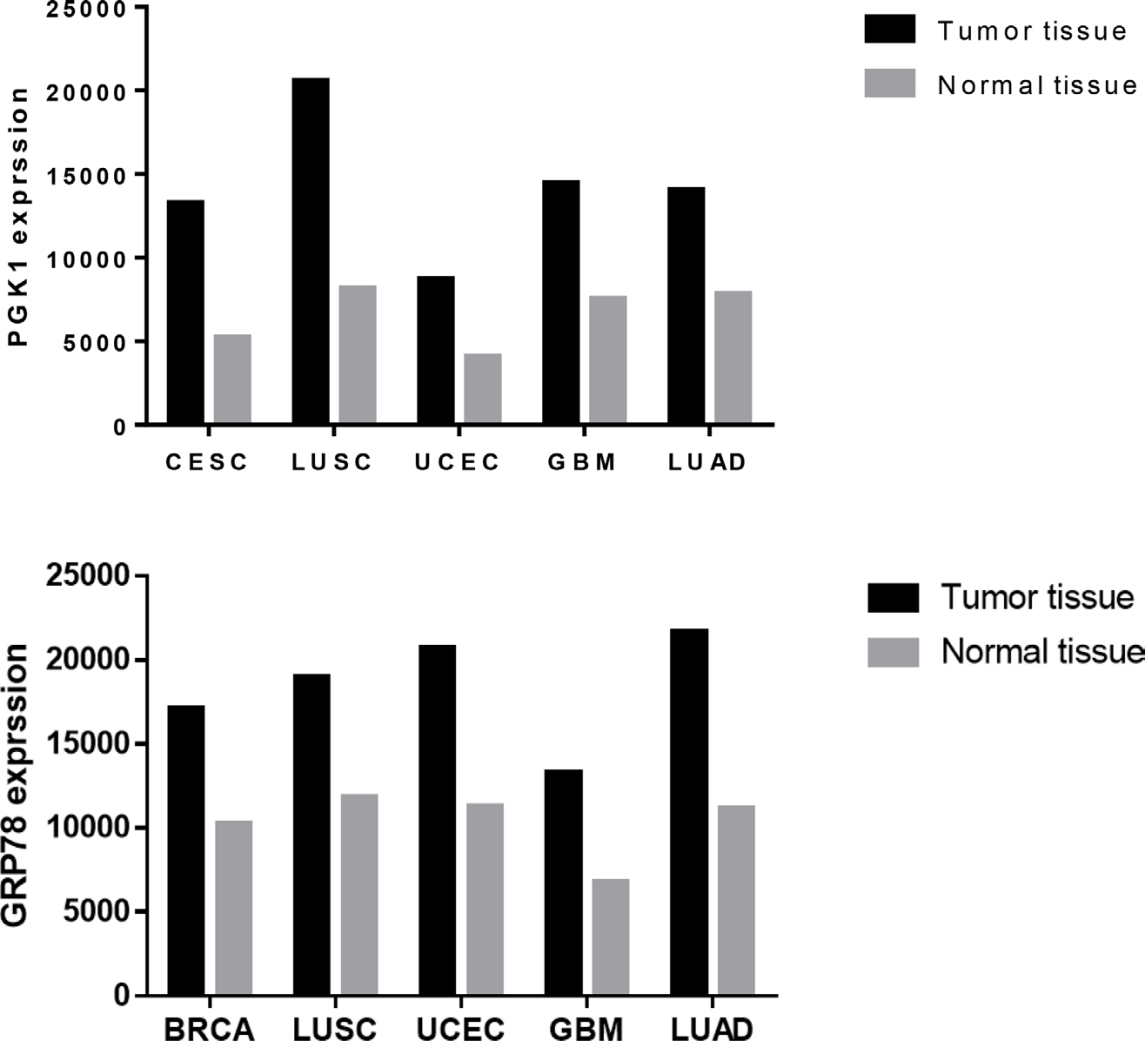
PGK1 and GRP78 expression in tumor tissues and normal tissues The data shown were obtained from the GCBI. BRCA: breast-invasive carcinoma, CESC: cervical squamous cell carcinoma and endocervical adenocarcinoma, GBM: glioblastoma multiforme, LUAD: lung adenocarcinoma, LUSC: lung squamous cell carcinoma, UCEC: uterine corpus endometrial carcinoma.

Accumulated evidence demonstrates that aberrant glucose metabolism, termed the “Warburg effect,” in cancer cells is closely associated with malignant phenotypes [4]. According to the biological information database String (Figure 1), we looked at the related genes in a network of glucose metabolism: PGAM2, TPI1, PGAM1, GAPDHS, GAPDH, ENO3, ENO1, ENO1, ENO1, ENO1, HSPA5 (GRP78), CANX and PGK1. In the preliminary experiment, we tested the proteins expressed from the above genes in the clinicopathological tissue samples and found that the PGK1 and GRP78 was highly expressed in the endometrial carcinoma samples compared with the normal endometrium samples, which is consistent with data obtained from the GCBI (Figure 2).

PGK1, the first ATP-generating enzyme in the glycolytic pathway, catalyzes the transfer of the high-energy phosphate from the 1-position of 1,3-bisphosphoglycerate (1,3-BPG) to ADP, which leads to the generation of 3-phosphoglycerate (3-PG) and ATP [5]. PGK1 expression is upregulated in human breast cancer, pancreatic ductal adenocarcinoma, radio-resistant astrocytoma, metastatic gastric cancer, and hepatocellular carcinoma cells [6–10]. However, no reports have described the role of PGK1 in endometrial carcinoma.

GRP78, a molecular chaperone in the endoplasmic reticulum (ER), is also found in the tumor cell plasma membrane, cytoplasm, mitochondria, nucleus, and in cellular secretions [11, 12]. GRP78 protein is usually highly induced in poorly perfused solid tumors by microenvironmental factors, including hypoxia, acidosis, and glucose deprivation. High levels of GRP78 contribute to the acquisition of phenotypic cancer hallmarks, including apoptosis resistance, immune escape, metastasis, and angiogenesis [13]. Recent research showed high expression of GRP78 to be associated with endometrial carcinoma [14, 15], However, whether GRP78 is involved in the clinicopathological characteristics and prognosis of endometrial carcinoma remains to be addressed.

## RESULTS

### PGK1 expression in normal endometria and endometrial carcinoma

**Figure 3:**
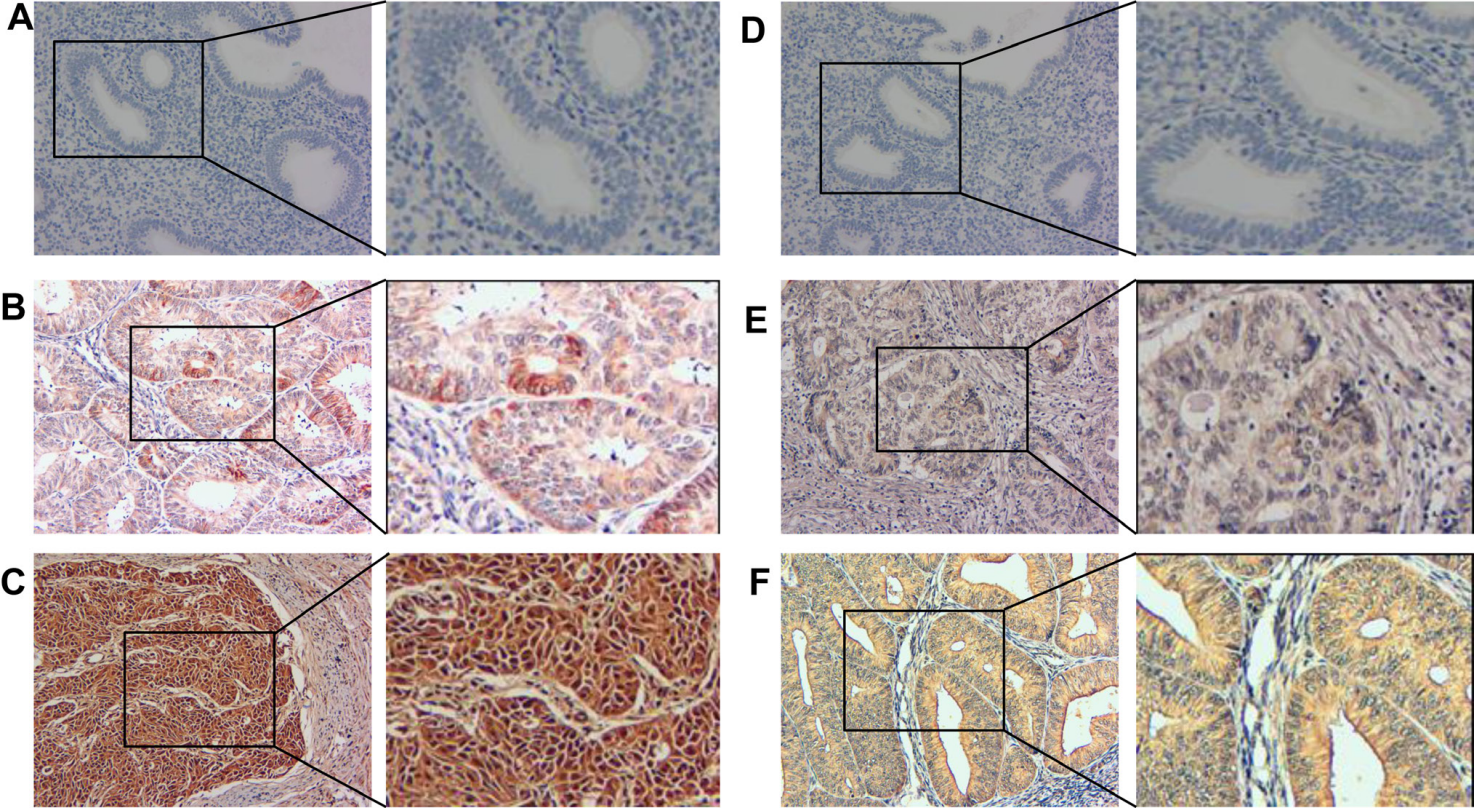
PGK1 and GRP78 expression in endometrial carcinoma and normal endometrial tissues were examined by immunohistochemistry Negative expression of PGK1 (**A**) and GRP78 (**D**) was demonstrated in a normal endometrial sample (200×). Light yellow PGK1 (**B**) and GRP78 (**E**) staining was observed in the cytoplasm of a stage I endometrial carcinoma case (200×). Brown PGK1 (**C**) staining was observed in the cytoplasm and nucleus of a stage II–III endometrial carcinoma case (200×), and brown GRP78 (**F**) staining was observed in the cytoplasm of a stage II–III endometrial carcinoma case.

We measured PGK1 protein-expression levels and localization in 130 endometrial carcinoma samples and 30 normal endometrial tissues by immunohistochemical staining (Figure 3). PGK1 expression was observed mostly in the cytoplasm of normal tissues and both in the cytoplasm and nucleus of tumor cells. In FIGO stages II–III, intense nuclear staining and weak cytoplasmic staining were observed. In addition, the PGK1 protein was highly expressed in 44.6% (58/130) of endometrial carcinoma samples, compared with only 10.0% (3/30) of normal samples, which was significantly lower than that in the endometrial carcinoma samples (*P* < 0.001) (Table 1).

**Table 1:** Expression levels of PGK1 and GRP78 in normal endometrial tissues and endometrial carcinoma samples

Variables	PGK1 (%)				GRP78 (%)			
	*N*	Low	High	*P*	*N*	Low	High	*P*
Tumor	130	72 (55.4)	58 (44.6)	0.000	130	70 (53.8)	60 (46.2)	0.003
Normal	30	27 (90.0)	3 (10.0)		30	25 (83.3)	5 (16.7)	

### GRP78 expression in normal endometrium and endometrial carcinoma

We measured the expression and subcellular localization of the GRP78 protein in 30 normal endometrial tissue samples and 130 endometrial carcinoma samples by immunohistochemical staining (Figure 3). GRP78 immunohistochemical staining was predominantly localized to the cytoplasm of noncancerous and neoplastic tissues. Moreover, we observed that the GRP78 protein was highly expressed in 46.2% (60/130) of endometrial carcinoma samples. In comparison, only 16.7% (5/30) of normal endometrial samples had high GRP78 protein expression, which was significantly lower than that in the endometrial carcinoma samples (*P* = 0.003) (Table 1).

### Association between clinicopathological characteristics and PGK1 expression in endometrial carcinoma patients

As shown in Table 2, PGK1 overexpression was positively correlated with the tumor clinical stage (I vs. II–III; *P* < 0.001), pathological type (adenocarcinoma vs. others; *P* = 0.007), histological grade (G1 vs. G2 vs. G3; *P* = 0.002), and lymph node metastasis (negative vs. positive; *P* < 0.001). Furthermore, we did not find significant differences between PGK1 expression and patient age, depth of myometrial invasion, or menopausal status in the 130 endometrial carcinoma cases.

**Table 2:** Correlations between PGK1 and GRP78 expression and clinicopathological parameters

Characteristics	PGK1 (%)				GRP78 (%)			
	*N*	Low	High	*P*	*N*	Low	High	*P*
Age								
<50	43	22 (51.2)	21 (48.8)	0.575	43	21 (48.8)	22 (51.2)	0.458
≥50	87	50 (57.5)	37 (42.5)		87	49 (56.3)	38 (43.7)	
Pathological type								
Adenocarcinoma	100	62 (62.0)	38 (38.0)	0.007	100	60 (60.0)	40 (40.0)	0.0125
Others	30	10 (33.3)	20 (66.7)		30	10 (33.3)	20 (66.7)	
Histological grading								
G1	56	37 (66.1)	19 (33.9)	0.002	56	36 (64.3)	20 (35.7)	0.019
G2	58	31 (53.4)	27 (46.6)		58	30 (51.7)	28 (48.3)	
G3	16	4 (25.0)	12 (75.0)		16	4 (25.0)	12 (75.0)	
Depth of myometrial invasion								
<50%	92	55 (59.8)	37 (40.2)	0.126	92	52 (56.5)	40 (43.5)	0.439
≥50%	38	17 (44.7)	21 (55.3)		38	18 (47.4)	20 (52.6)	
Lymph node status								
Negative	112	71 (63.4)	41 (36.6)	0.000	112	69 (61.6)	43 (38.4)	0.000
Positive	18	1 (5.6)	17 (94.4)		18	1 (5.6)	17 (94.4)	
FIGO stage								
I	99	68 (68.7)	31 (31.3)	0.000	99	64 (64.6)	35 (35.4)	0.000
II–III	31	4 (12.9)	27 (87.1)		31	6 (19.4)	25 (80.6)	
Menopausal status								
Premenopausal	70	39 (55.7)	31 (44.3)	1.000	70	37 (52.9)	33 (47.1)	0.861
Postmenopausal	60	33 (55.0)	27 (45.0)		60	33 (55.0)	27 (45.0)	

FIGO, the International Federation of Gynecology and Obstetrics; G1, well differentiated; G2, moderately differentiated; G3 poorly differentiated. *P* values were determined by the χ^2^ test. The other pathological types include uterine papillary serous carcinoma, clear cell carcinoma, adenosquamous carcinoma, among others.

### Association between the clinicopathological characteristics and GRP78 expression in endometrial carcinoma patients

Clinicopathological characteristics and GRP78 expression levels in individuals with endometrial carcinoma are summarized in Table 2. We observed that the expression level of GRP78 was positively correlated with FIGO stage (I vs. II–III; *P* < 0.001), pathological type (adenocarcinoma vs. others; *P* = 0.0125), histological grade (G1 vs. G2 vs. G3; *P* = 0.019), and lymph node metastasis (negative vs. positive; *P* < 0.001) in endometrial carcinoma patients. However, we did not find a significant association of GRP78 expression levels with patient age, depth of myometrial invasion, or menopausal status in the 130 endometrial carcinoma cases.

### Association between PGK1 and GRP78 expression in endometrial carcinoma patients

As shown in Table 3, Spearman’s test demonstrated that PGK1 expression was positively correlated with GRP78 expression in the endometrial carcinoma patients (*P* < 0.001). As summarized in Table 4, the association between the co-expression levels of both proteins with the FIGO stage (I vs. II–III; *P* < 0.001), pathological type (adenocarcinoma vs. others; *P* = 0.005), histological grade (G1 vs. G2 vs. G3; *P* = 0.010), and lymph node metastasis (negative vs. positive; *P* < 0.001) was significant in endometrial carcinoma patients. However, significant association were not observed with the patient age, depth of myometrial invasion, or menopausal status.

**Table 3:** Correlation between the expression of PGK1 and GRP78 in endometrial carcinoma patients

Variables	PGK1 (%)				
	*N*	Low expression	High expression	*r*	*P*
GRP78					
Low expression	70	63 (90.0)	7 (10.0)	0.752	<0.001
High expression	60	9 (15.0)	51 (85.0)		

*P* values were determined by the Pearson test.

**Table 4:** Co-expression of PGK1 and GRP78 in endometrial carcinoma

Characteristics	PGK1 &GRP78			
	*N*	LL	HH	*P*
Age				
<50	40	20 (50.0)	20 (50.0)	0.435
≥50	74	43 (58.1)	31 (41.9)	
Pathological type				
Adenocarcinoma	90	56 (62.2)	34 (37.8)	0.005
Others	24	7 (29.2)	17 (70.8)	
Histological grading				
G1	51	34 (66.7)	17 (33.3)	0.010
G2	49	26 (53.1)	23 (46.9)	
G3	14	3 (21.4)	11 (78.6)	
Depth of myometrial invasion				
<50%	80	42 (52.5)	38 (47.5)	0.414
≥50%	34	21 (61.8)	13 (38.2)	
Lymph node status				
Negative	98	63 (64.3)	35 (35.7)	0.000
Positive	16	0 (0.0)	16 (100.0)	
FIGO stage				
I	87	60 (69.0)	27 (31.0)	0.000
II-III	27	3 (11.1)	24 (88.9)	
Menopausal status				
Premenopausal	60	33 (55.0)	27 (45.0)	0.551
Postmenopausal	54	30 (55.6)	24 (44.4)	

FIGO, the International Federation of Gynecology and Obstetrics; G1, well differentiated; G2, moderately differentiated; G3 poorly differentiated; HH, high expression of PGK1 and GRP78; LL, low expression of PGK1 and GRP78. *P* values were determined by the χ^2^ test.

### High expression of PGK1 and GRP78 is associated with overall survival time in endometrial carcinoma

**Figure 4:**
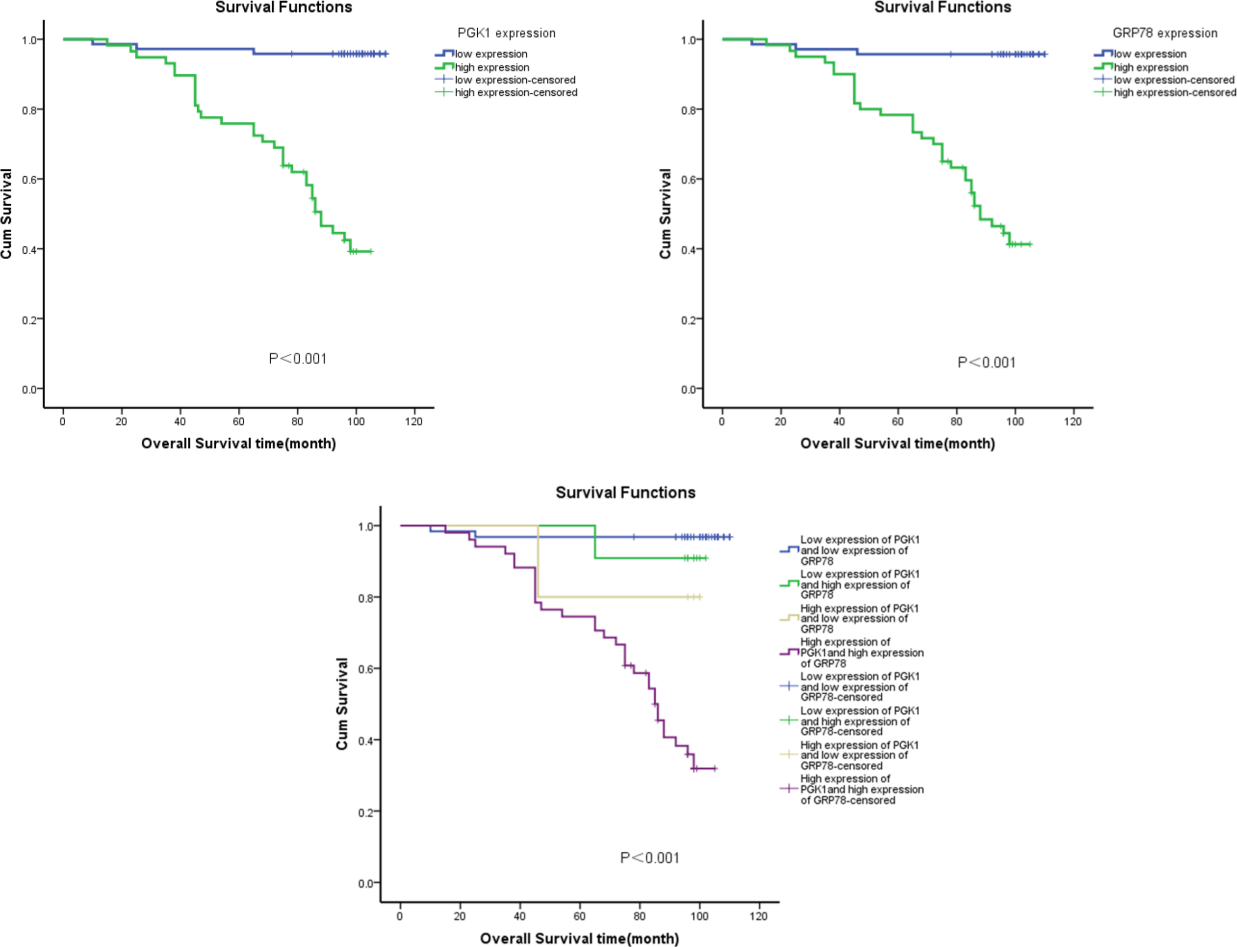
Kaplan–Meier survival analysis of overall survival duration in 130 endometrial carcinoma patients according to PGK1 and GRP78 protein expression The log-rank test was used to calculate *P* values.

To investigate the prognostic value of PGK1 and GRP78 expression for endometrial carcinoma, we assessed the association between PGK1 and GRP78 expression levels and patient survival using Kaplan–Meier analysis with the log-rank test. In 130 endometrial carcinoma cases with prognosis information, the levels of PGK1 and GRP78 expression significantly correlated with overall survival. Patients with high PGK1 and GRP78 expression had worse prognoses than those with low expression of these proteins (Figure 4) (*P* < 0.001).

### High expression of GRP78 is an independent prognostic factor for endometrial carcinoma patients

Univariate analyses showed that FIGO stage, histological grade, lymph node status, high PGK1 expression, high GRP78 expression, postoperative irradiation, and postoperative chemotherapy were also significantly correlated with patient survival ( *P* < 0.001, *P* = 0.001, *P* < 0.001, *P* < 0.001, *P* < 0.001, *P* = 0.042, and *P* = 0.003, respectively). To determine whether PGK1 and GRP78 are independent prognostic factors for endometrial carcinoma, we performed multivariate analysis of PGK1 and GRP78 protein expression levels, adjusted for career, FIGO stage, histological grading, lymph node status, postoperative irradiation, and postoperative chemotherapy of endometrial carcinoma patients. These results showed that the level of GRP78 expression was an independent prognostic factor for endometrial carcinoma patients (*P* = 0.004), whereas PGK1 expression was not (*P* = 0.077) (Table 5).

**Table 5:** Summary of univariate and multivariate Cox regression analysis of overall survival duration

Parameter	Univariate analysis			Multivariate analysis		
	*P*	HR	95% CI	*P*	HR	95% CI
Age						
<50 versus ≥50	0.220	1.514	0.780–2.938			
Family history of tumor						
Negative versus positive	0.950	0.972	0.405–2.336			
Health insurance						
No versus yes	0.166	0.613	0.306–1.226			
Menopausal status						
Premenopausal versus postmenopausal	0.483	1.264	0.657–2.430			
Complications						
With versus without	0.233	1.497	0.772–2.904			
FIGO stage						
I versus II + III	<0.001	12.882	6.318–26.266	0.001	7.085	2.308–21.750
Histological grade						
G1 versus G2 versus G3	0.001	2.335	1.444–3.776	0.001	2.509	1.471–4.277
Lymph node status						
Negative versus positive	<0.001	14.899	7.264–30.562	0.010	4.497	1.424–14.199
Depth of myometrial invasion						
<50% versus ≥50%	0.821	1.085	0.534–2.206			
GRP78 expression						
Low versus high	<0.001	18.067	5.516–59.169	0.004	7.274	1.869–28.309
PGK1 expression						
Low versus high	<0.001	19.672	6.005–64.445	0.077	3.250	0.882–11.977
Postoperative irradiation						
Yes versus no	0.042	2.141	1.029–4.453	0.797	1.126	0.456–2.785
Postoperative chemotherapy						
Yes versus no	0.003	2.722	1.397–5.307	0.797	0.884	0.345–2.266
Postoperative hormone therapy						
Yes versus no	0.125	0.573	0.282–1.166			

### Receiver operating characteristic (ROC) analysis of the combined expression of PGK1 and GRP78 for prognosis

**Figure 5:**
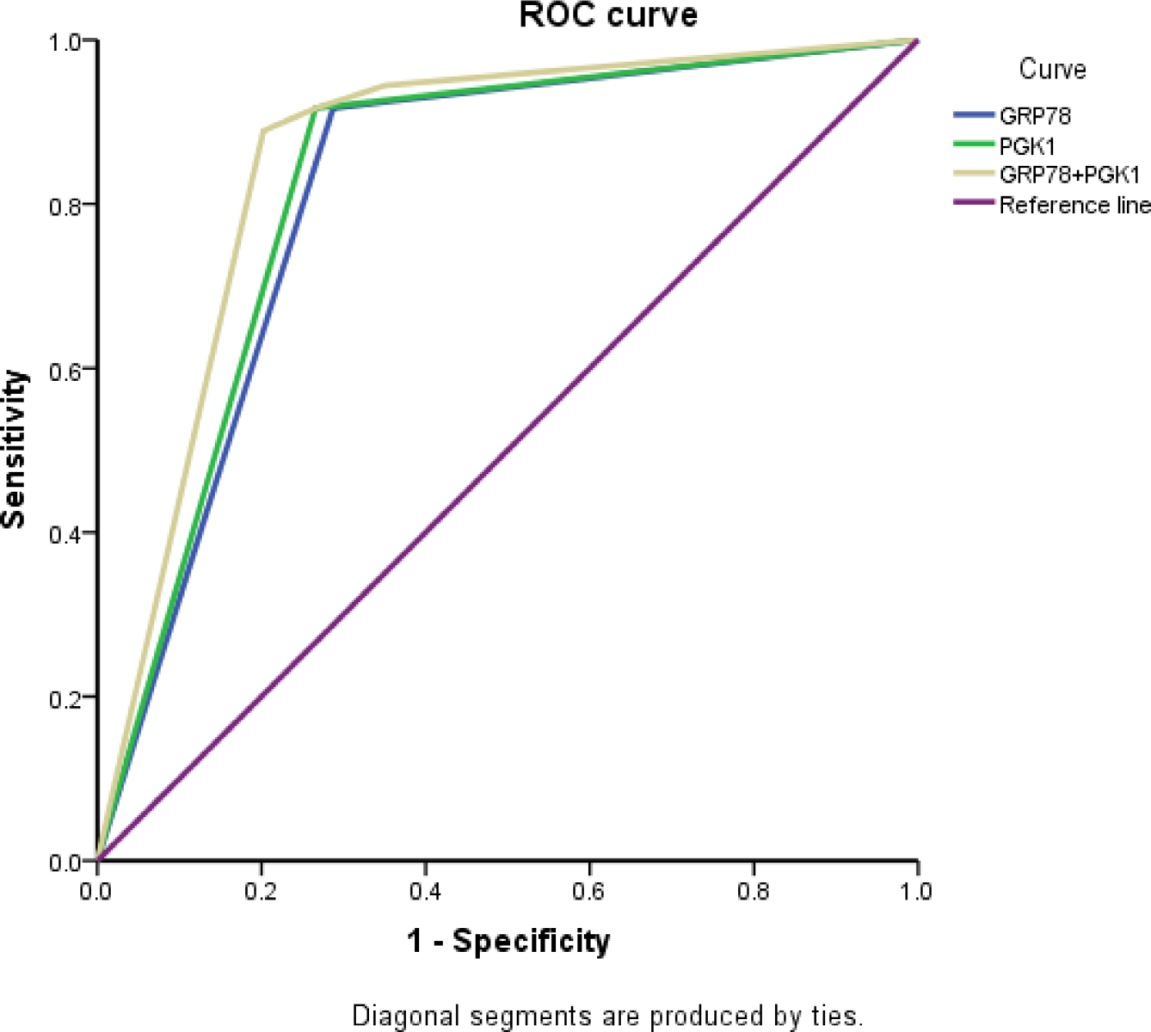
ROC analysis for the predictive value of PGK1 and GRP78 expression in patients with endometrial carcinoma

ROC analysis was performed to evaluate the efficacy of the protein markers. The area under the curve (AUC) was 0.815 using GRP78 expression as a predictive model (95% CI: 0.737–0.893; *P* < 0.001) and 0.825 using PGK1 expression (95% CI: 0.749–0.902; *P*  <  0.001). To generate a more sensitive predictive model for patient outcomes, we combined PGK1 expression and GRP78 expression to create a prognostic scoring system. The combination improved the prognostic value; the AUC was 0.858 (95% CI: 0.788–0.927; *P*  <  0.001), which was larger than that of GRP78 expression or PGK1 expression alone (Figure 5). The C-index was 0.815 when assessed by GRP78 expression alone, and it increased to 0.858 when PGK1 expression was added.

## DISCUSSION

In this study, we systematically examined the expression levels of PGK1 and GRP78 in normal endometrium and endometrial carcinoma tissues, and analyzed the correlations between the expression of these markers and clinicopathological parameters.

PGK1 is the first ATP-generating enzyme in the glycolytic pathway. It catalyzes the reversible conversion of 1,3-BPG and ADP to 3-phosphoglycerate and ATP, respectively [18–21]. Many studies have shown that PGK1 acts as a protein kinase in coordinating glycolysis and autophagy, which is instrumental in cancer metabolism and tumorigenesis [22–25]. To our knowledge, this is the first study to measure PGK1 expression by immunohistochemical staining in endometrial carcinoma and normal endometrium tissues, and demonstrate an association between PGK1 expression and the clinical features of endometrial carcinoma. We found that PGK1 expression in endometrial carcinoma tissues was higher than that in normal endometrium tissues, consistent with earlier studies [26, 27]. Here, significant associations between high PGK1 expression and FIGO stage, histological grade, pathological type, and lymph node metastasis were identified. Similar to a report on gallbladder cancer [28], we further demonstrated that high PGK1 expression was associated with poor prognosis in endometrial carcinoma patients.

GRP78 resides in the ER, and its expression is induced by an unfolded-protein response triggered under many kinds of cellular stresses. GRP78 can also act as an anti-apoptotic factor by protecting cells against ER stress-induced cell death [29, 30]. Recent research has shown that GRP78 is involved in the biological processes of a variety of tumors [31–33]. In our study, we first examined the expression of GRP78 in Chinese endometrial carcinoma patients and found that GRP78 was highly expressed in endometrial carcinoma tissues compared with normal endometrial tissues and that this high expression was associated with the histological grade, lymph node status, FIGO stage, and overall survival time of the endometrial carcinoma patient, consistent with previous studies [34, 35]. Furthermore, we identified GRP78 as an independent prognostic factor for endometrial carcinoma patients.

Ingenuity pathway analysis (IPA) is used to study proteomic data. Xu *et al.* found that PGK1 and GRP78 are associated in a network by IPA and that they are negatively correlated to type-5 17 beta-hydroxysteroid dehydrogenase in breast cancer cell viability and proliferation [36]. To our knowledge, this is the first study to determine the association between PGK1 and GRP78 expression in endometrial carcinoma. Given that Spearman’s test showed that the PGK1 and GRP78 expression levels were statistically correlated, we examined the association between protein co-expression and clinicopathological parameters. We found that the co-expression of both proteins correlated significantly with the pathological type, histological grade, lymph node status, and FIGO stage. Furthermore, ROC analysis showed that combined ROC analysis of PGK1 and GRP78 could better determine the prognosis of endometrial carcinoma patients.

## CONCLUSIONS

In summary, we have shown for the first time that high expression of PGK1 and GRP78 might be involved in the clinical progression and poor prognosis of endometrial carcinoma. Furthermore, our results suggest that high expression of PGK1 and GRP78 might serve as a new clinically significant biomarker for endometrial carcinoma prognosis. Combined PGK1 and GRP78 can improve the assessment with the prognosis of endometrial carcinoma patients. Owing to the limited sample size of patients in our study, further investigations are needed to confirm these findings and establish the role of PGK1 and GRP78 as a reliable clinical predictor for endometrial carcinoma outcomes. Endometrial carcinoma is a multigene-regulatory disease, and we investigated the potential for tumor diagnosis, prognosis, and treatment from a multigene perspective. Our findings suggested that PGK1 expression and GRP78 expression are statistically correlated with each other, and that the inhibition of PGK1 and GRP78 activation could be an effective approach for slowing the disease, providing the foundation for the application of PGK1 and GRP78 inhibitors as a therapeutic strategy in the future.

## MATERIALS AND METHODS

### Sample collection

From 2003 to 2008, formalin-fixed paraffin-embedded samples of 30 normal endometrial tissue samples and 130 endometrial carcinoma samples were obtained from the Third Affiliated Hospital of Guangzhou Medical School, Guangzhou City, China. All endometrial carcinoma patients underwent surgery, which consisted of diagnostic curettage, total hysterectomy, bilateral salpingo-oophorectomy, and pelvic and para-aortic lymph node sampling when necessary. No patient underwent chemotherapy or radiotherapy before surgery. In the 130 endometrial carcinoma cases, the median age of the patients was 49.6 years (range, 32–82). The clinical follow-up time of patients ranged from 45 to 110 months. Prior consent from the patients and approval from the Ethics Committees of this hospital were obtained before using these clinical materials for research purposes. All specimens had confirmed pathological diagnosis and were staged according to the FIGO 2009 guidelines.

### Immunohistochemistry

Two paraffin-embedded sections (3 µm) each from 130 endometrial carcinoma samples and 30 normal endometrium specimens were deparaffinized in 100% xylene and rehydrated in a descending ethanol series (100%, 90%, 80%, and 70% ethanol) and water according to standard protocols. Heat-induced antigen retrieval was performed in 10 mM citrate buffer for 2 min at 100°C. Endogenous peroxidase activity and nonspecific antigens were blocked with peroxidase blocking reagent containing 3% hydrogen peroxide and serum, followed by incubation with goat anti-human polyclonal PGK1 antibody (1:50) (ProteinTech Group, Catalog number 17811-1-AP) and GRP78 (1:50) (ProteinTech Group, Catalog number 11587-1-AP), respectively, overnight at 4°C. Sections were washed and incubated with biotin-labeled rabbit anti-goat antibody for 10 min at room temperature, and were subsequently incubated with streptavidin-conjugated horseradish peroxidase (Maixin, Inc., China, Guangzhou). The peroxidase reaction was developed by using 3,3′-diaminobenzidine (DAB) chromogen solution in DAB buffer substrate. Sections were visualized with DAB, counterstained with hematoxylin, mounted in neutral gum, and analyzed by bright-field microscopy.

### Evaluation of staining

Immunohistochemically stained tissue sections were reviewed and scored separately by 2 pathologists, who were blinded to the clinical parameters. The staining intensity was scored as previously described [16, 17]. The extent of the staining, defined as the percentage of positively stained area of tumor cells or normal endometrial cells in relation to the whole tissue area, was scored on a scale of 0 to 4 as follows: 0, <10%; 1, 10–25%; 2, 26–50%; 3, 50–75%; and 4, >76%. The sum of the staining intensity and staining-extent scores was used as the final staining score for PGK1 or GRP78 (0–7). For statistical analysis, final staining scores of 0–5 and 6–7 were considered to reflect low and high expression, respectively.

### Statistical analyses

SPSS 21.0 software was applied to perform all statistical analyses. The χ^2^ test was used to verify the relationship between the clinicopathological characteristics and the expression of the 2 oncogenes. Spearman’s test was performed using the H-scores to examine the pairwise comparisons between the 2 oncogenes. A value of less than 0.05 was considered statistically significant.
